# Association of Lower Extremity Muscle Strength and Function with Renal Resistive Index in Individuals with and without Chronic Kidney Disease

**DOI:** 10.3390/geriatrics8060118

**Published:** 2023-12-03

**Authors:** Natsumi Nishitani, Keisei Kosaki, Shoya Mori, Masahiro Matsui, Takeshi Sugaya, Makoto Kuro-o, Chie Saito, Kunihiro Yamagata, Seiji Maeda

**Affiliations:** 1 Graduate School of Comprehensive Human Sciences, University of Tsukuba, Ibaraki 305-8577, Japan; natsu.21kyk@gmail.com; 2 Institute of Health and Sport Sciences, University of Tsukuba, Ibaraki 305-8577, Japan; sho33mori@gmail.com (S.M.); masahiromatsui0708@gmail.com (M.M.); seiji.maeda@waseda.jp (S.M.); 3 Broad Bean Science Incorporation, Tochigi 329-0498, Japan; 4 Institute of Health and Sports Science & Medicine, Juntendo University, Chiba 270-1695, Japan; 5 Division of Nephrology and Hypertension, Department of Internal Medicine, St. Marianna University School of Medicine, Kawasaki 216-8511, Japan; takeshi-sugaya@marianna-u.ac.jp; 6 Division of Anti-Aging Medicine, Center for Molecular Medicine, Jichi Medical University, Tochigi 329-0498, Japan; mkuroo@jichi.ac.jp; 7 Faculty of Medicine, University of Tsukuba, Ibaraki 305-8577, Japan; chie.saito@md.tsukuba.ac.jp (C.S.); k-yamaga@md.tsukuba.ac.jp (K.Y.); 8 R&D Center for Smart Wellness City Policies, University of Tsukuba, Ibaraki 305-8577, Japan; 9 Faculty of Sport Sciences, Waseda University, Saitama 359-1192, Japan

**Keywords:** renal resistive index, lower extremity, chronic kidney disease, renal flow pulsatility

## Abstract

Age-related loss of lower extremity muscle strength is pronounced in individuals with chronic kidney disease (CKD). In contrast, an increase in intrarenal flow pulsatility results in initial age-related changes in renal hemodynamics, leading to the development of CKD. To date, it remains unclear whether lower extremity muscle strength determines elevated renal flow pulsatility. This study aimed to determine the association of lower extremity muscle strength and function with intrarenal hemodynamics in individuals with and without CKD. One hundred seventy-six individuals without CKD (aged 63 ± 9 years) and 101 individuals with CKD (aged 66 ± 8 years) were included in this study. Using Doppler ultrasound, the renal resistive index (RI) was measured as a parameter of renal hemodynamics. Knee extensor muscle strength (KES), gait speed (GS), and the 30 s chair stand test (30s-CST) were used to measure lower extremity muscle strength and function. Multivariate analyses showed that GS and 30s-CST scores were independent determinants of renal RI, whereas the KES score was not associated with renal RI in individuals with and without CKD. In the two-way analysis of covariance, renal RI was the highest in individuals with CKD who had lower KES, GS, and 30s-CST scores. Reduced lower extremity muscle strength and function are independent determinants of elevated renal flow pulsatility in individuals with and without CKD.

## 1. Introduction

The kidneys are high-flow, low-resistance organs that receive approximately 20–25% of the cardiac output at rest [1]. Furthermore, the kidneys have numerous short arteries branching from major arteries, referred to as “strain vessels” [2]. While these kidney characteristics are essential for maintaining normal glomerular filtration, they also render them vulnerable to the mechanical stress of arterial pulsatility [3]. Continuous exposure of renal microcirculation to high flow pulsatility can lead to renal dysfunction and damage [4,5]. Therefore, close monitoring of the pulsatile components in the kidneys may contribute to the early detection of progressive renal dysfunction and damage in clinical settings.

Renal duplex ultrasonography has been used for noninvasive evaluation of blood flow pulsatility in the kidneys [6]. Previous studies have demonstrated that the renal resistive index (RI) derived from the pulsatile flow-velocity waveform is associated with several histological and biochemical findings of renal microvascular damage, such as peritubular capillary loss [7]. Moreover, high renal RI is associated with cardiovascular events and mortality in patients with hypertension, diabetes, and chronic kidney disease (CKD) [8,9,10,11,12]. Thus, the number of studies highlighting the clinical significance of renal RI is increasing. However, evidence regarding the determinants of renal RI values remains insufficient. Elucidating the determinants of renal RI is crucial when designing intervention strategies targeting renal flow pulsatility.

Our previous study reported that advancing age is associated with a progressive elevation of renal RI, which manifests during middle age and accelerates later in life [13]. Additionally, it has been demonstrated that renal RI is significantly influenced by extrarenal hemodynamic factors, such as central pulse pressure and stiffness [14]. In addition to these physiological determinants, we have reported a significant correlation between renal RI and handgrip strength in middle-aged and older individuals with normal kidney function [15]. In this study, the reduced handgrip strength was related to the elevated renal RI, independent of age and central hemodynamics, suggesting that reduced muscular strength may be a determinant of increased renal RI [15]. It has been shown that reduced muscle strength and function were associated with an increased risk of cardiovascular disease (CVD), such as coronary heart disease and stroke [16]. Reduced muscle strength and function can emerge due to multi-factorial mechanisms such as inflammation, oxidative stress, and metabolic disorders, all of which can also lead to vascular dysfunction [16]. We consider that these previous findings can extrapolate into the association of muscle strength and function with renal hemodynamics. However, it is worth noting that prior research only assessed handgrip strength, mainly reflecting upper limb strength, as a measure of muscular strength. It is essential to investigate the relationship between various measures of muscular strength, including lower extremity muscle strength, and renal RI to assert that muscular strength is a determinant of renal RI. Furthermore, there is room for investigation regarding whether a similar relationship is observed in a population with CKD. Therefore, the present study aimed to investigate the cross-sectional association of lower extremity muscle strength and function with renal flow pulsatility (i.e., renal RI) in individuals with and without CKD. We hypothesized that individuals with CKD who have decreased lower extremity muscle strength and function would have high renal flow pulsatility.

## 2. Materials and Methods

### 2.1. Participants

**Figure 1:**
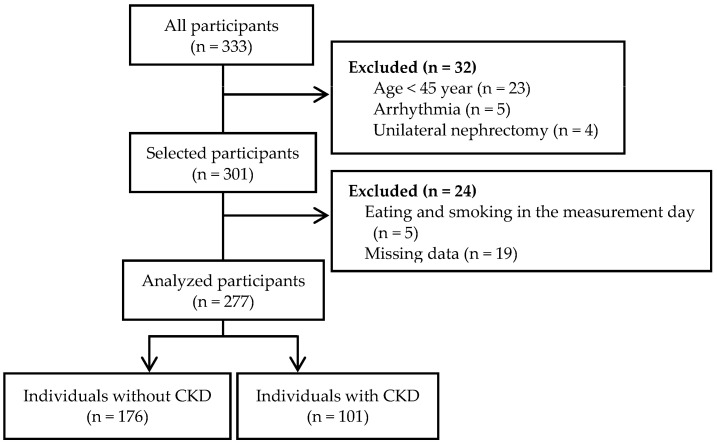
Flow diagram.

In this study, 333 adults were recruited through local newspaper advertisements, flyers, and the Department of Nephrology of the University of Tsukuba Hospital. The exclusion criteria were age < 45 years (n = 23), arrhythmia (n = 5), and unilateral nephrectomy (n = 4). Participants who ate or smoked on the morning of the measurement day (n = 5) or had missing data (n = 19) were also excluded from the analysis. The final sample included 277 participants comprising 176 individuals without CKD and 101 individuals with CKD who were diagnosed according to standard criteria [17]: estimated glomerular filtration rate (eGFR) < 60 mL/min/1.73 m^2^ and/or urinary albumin creatinine ratio (ACR) ≥ 30 mg/g (Figure 1). Although the diagnosis of CKD usually requires that these conditions persist for at least 3 months, in the current study, eGFR and urinary ACR were measured at a single time point according to a previous report [18]. The participants were required to avoid engaging in vigorous exercise and ingesting alcohol and caffeine 24 h before the measurement. All measurements, except for the lower extremity muscle strength and function tests, were performed in the morning after 12 h of overnight fasting. This study was approved by the Ethics Committee of the University of Tsukuba Hospital (approval no. H30-161). The study conformed to the principles outlined in the Declaration of Helsinki, and written informed consent was obtained from all participants.

### 2.2. Measurements

#### 2.2.1. Renal Doppler Ultrasound

Renal blood flow velocity (BFV) in the intrarenal segmental arteries of each kidney was measured using Doppler ultrasonography with a 3.5 MHz convex array probe (Noblus C25, Hitachi Aloka Medical Ltd., Tokyo, Japan). All renal BFV measurements were performed with the focal zone set to the depth of the target artery, and the probe insonation angle to the target artery was set to less than 60°. Because breathing changes the position of the kidneys, the participants were instructed to hold their breath for 10–15 s during renal BFV measurements. Renal BFV was measured thrice (three segmental arteries in each kidney). Renal RI was calculated from renal flow pulsatility indices using the following equation: (peak systolic BFV—end-diastolic BFV)/peak systolic BFV [19]. The average of three measurements for each kidney (more than six times in total) was used for statistical analysis. The renal BFV measurement was performed by a well-trained examiner who understands the international standard procedure at a single center. This reduced the inter-operator error in the renal flow pulsatility assessment. Additionally, we presented the number of participants who had a renal RI over 0.70 because 0.70 was established as a cutoff value of renal RI to predict future clinical events [8,10].

#### 2.2.2. Lower Extremity Muscle Strength and Function

We evaluated three indices that have been reported to be useful in assessing lower extremity muscle strength and function [20,21,22,23]. Knee extensor muscle strength (KES) and the 30 s chair stand test (30s-CST) were evaluated for lower extremity muscle strength, and gait speed (GS) was evaluated for lower extremity function. KES was measured using a handheld dynamometer (μTas; Anima, Tokyo, Japan). The handheld dynamometer was fixed to the leg, directly opposing the leg motion. Measurements were obtained from subjects sitting on the bed with their legs perpendicular to the floor and knees flexed approximately 90°. The participants gradually increased force to maximum extensions and then maintained a static state for approximately 3 s, during which the maximum force was noted. Isometric knee extension strength is strongly influenced by body mass; therefore, the values were divided by body mass [24,25]. The averages of the left and right maximum values were used for analysis [26]. GS was calculated for each participant using distance in meters and time in seconds. The walking distance was 10 m, and the participants were instructed to walk as fast as possible from a standing start position [27]. Further, 30s-CST was measured using a chair with a height of 40 cm. The participants repeated the action, rose to a complete stand, and returned to their initial seated position. The number of complete stands performed within 30 s was used for analysis [28,29].

#### 2.2.3. Biochemical Measurements

Blood samples were collected from antecubital veins. Serum samples were used to measure high-density lipoprotein (HDL) cholesterol, low-density lipoprotein (LDL) cholesterol, triglyceride, fasting blood glucose, creatinine, and cystatin C levels. Hemoglobin A1c (HbA1c) levels were measured in whole blood samples. Two eGFR values were calculated from serum creatinine and cystatin C levels [30,31], and the values were averaged to improve the accuracy of these estimates [32]. Urinary albumin and creatinine concentrations were measured in spot urine samples. The urinary albumin concentration was corrected for the urinary creatinine concentration to adjust for the effect of urine specific gravity.

#### 2.2.4. Covariates

Anthropometric measurements were performed, and body mass index (BMI) was calculated from height and weight in kg/m^2^. Brachial systolic blood pressure (SBP), brachial diastolic blood pressure (DBP), heart rate, and pulse wave velocity (PWV) were measured using a semiautomated vascular testing device (Form PWV/ABI, Colin Medical Technology, Aichi, Japan). Carotid arterial pressure waveform was measured using applanation tonometry sensors (TU-100; Colin Medical Technology, Aichi, Japan). The pressure waveform data were fed into SphygmoCor software version 8.2 (AtCor Medical, Sydney, Australia), and a generalized transfer function was applied to estimate the aortic pulse pressure [33]. The carotid-femoral PWV, indicating aortic stiffness, was calculated by dividing the distance between the two arterial sites (carotid and femoral) where the tonometry sensors were attached by the arterial pulse transit time [34]. The levels of physical activity (metabolic equivalents [METs]∙hour/week) in daily life were assessed using the International Physical Activity Questionnaire Short Version [35]. In this study, the underlying conditions were diagnosed when one of the following criteria was met: hypertension (brachial SBP: ≥140 mmHg, brachial DBP: ≥90 mmHg, and/or antihypertensive medication use), diabetes mellitus (fasting blood glucose: ≥126 mg/dL, HbA1c: ≥6.5%, and/or hypoglycemic medication use), and dyslipidemia (HDL cholesterol: <40 mg/dL, LDL cholesterol: ≥140 mg/dL, triglyceride: ≥150 mg/dL, and/or use of antidyslipidemic medication).

### 2.3. Statistical Analysis

Data are expressed as mean ± standard deviation, median with interquartile range, or frequencies and percentages, unless otherwise indicated. Statistical analyses were performed using SPSS Statistics software 28.0 (IBM Japan Ltd., Tokyo, Japan). Statistical significance was set at *p* < 0.05. The group differences between individuals with and without CKD were examined using the unpaired t-test, Mann–Whitney U test, or chi-square test. Univariate linear associations were assessed using Pearson’s correlation coefficients. Multiple linear regression analysis was used to estimate the regression coefficients (*β*) for the association of lower extremity muscle strength and function with renal RI in all participants, individuals without CKD, and individuals with CKD, separately. All models were adjusted for age, sex, BMI, eGFR, urinary ACR, comorbidities (hypertension, diabetes mellitus, and dyslipidemia), physical activity level, heart rate, aortic pulse pressure, and carotid-femoral PWV. Two-way analysis of covariance (ANCOVA) was performed to examine the interaction between lower extremity muscle strength and function measures (KES, GS, and 30s-CST) and CKD. Participants were allocated to one of four categories based on the median split of each lower extremity muscle strength measure and the presence of CKD. Covariates used in the multiple linear regression analysis were adjusted. Post hoc comparisons were corrected using Bonferroni’s method.

## 3. Results

Table 1 shows the characteristics of all participants. The mean age of all participants, those without CKD, and those with CKD were 64 ± 9 years, 63 ± 9 years, and 66 ± 8 years, respectively. There were no differences in height, BMI, KES score, physical activity levels, or prevalence of dyslipidemia between individuals with and without CKD. The proportion of women, eGFR values, GS, and 30s-CST scores were lower in individuals with CKD than in those without CKD. In contrast, age, body mass, urinary ACR, heart rate, brachial SBP, brachial DBP, mean arterial pressure, aortic pulse pressure, carotid-femoral PWV, renal RI, prevalence rates of hypertension and diabetes mellitus, and rate of medication use were higher in individuals with CKD than in those without CKD.

The renal RI for individuals without and with CKD by GFR stage is shown in Table 2. Among participants with eGFR stage 1, significant differences in renal RI were shown between those without CKD and those with CKD (*p* = 0.032). In participants with eGFR stage 2, renal RI did not differ significantly between those without CKD and those with CKD (*p* = 0.161).

**Figure 2:**
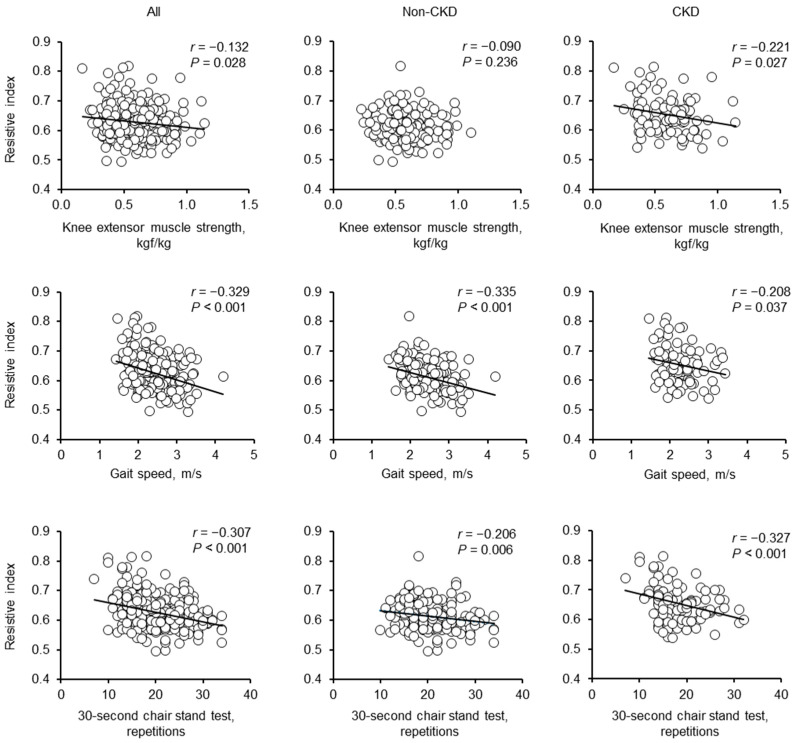
Univariate correlations between the renal RI and lower extremity muscle strength in individuals without CKD, individuals with CKD, and all participants.

As presented in Figure 2, renal RI was negatively correlated with KES, GS, and 30s-CST scores in all participants and individuals with CKD; however, in those without CKD, renal RI was negatively correlated with GS and 30s-CST scores but not significantly with the KES score. In all participants, renal RI positively correlated with age, BMI, urinary ACR, brachial SBP, aortic pulse pressure, and carotid-femoral PWV and negatively correlated with eGFR. In contrast, renal RI showed no significant correlation with heart rate, brachial DBP, mean arterial pressure, or physical activity levels. In adults without CKD, renal RI positively correlated with age, urinary ACR, brachial SBP, aortic pulse pressure, and carotid-femoral PWV and negatively correlated with eGFR (Table 3).

In contrast, renal RI showed no significant correlation with BMI, heart rate, brachial DBP, mean arterial pressure, or physical activity level. In individuals with CKD, renal RI was positively correlated with age, BMI, urinary ACR, aortic pulse pressure, and carotid-femoral PWV and negatively correlated with eGFR and brachial DBP. In contrast, renal RI showed no significant correlation with heart rate, brachial SBP, mean arterial pressure, or physical activity levels.

The results of the multiple linear regression analyses of renal RI are presented in Table 4 (all participants), Table 5 (individuals without CKD), and Table 6 (individuals with CKD). KES score was not significantly associated with renal RI after adjusting for covariates such as age and sex in all participants, adults without CKD, and patients with CKD (*β* = −0.003, *p* = 0.952; *β* = −0.097, *p* = 0.129; *β* = −0.093, *p* = 0.235, respectively). In contrast, GS and 30s-CST scores remained independent determinants of renal RI, even after adjusting for covariates.

**Figure 3:**
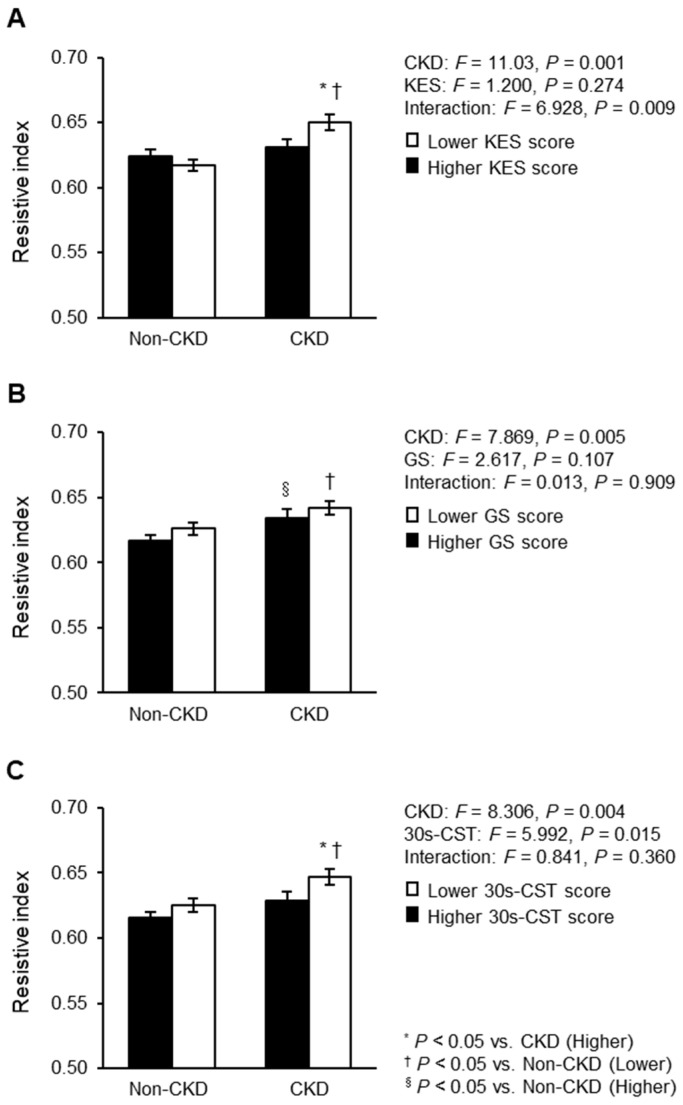
Renal RI compared between CKD status and lower extremity muscle strength: (**A**) KES; (**B**) GS; (**C**) 30s-CST. Mean ± standard error. These associations were adjusted for age, sex, BMI, comorbidities (hypertension, diabetes mellitus, and dyslipidemia), carotid-femoral PWV, aortic pulse pressure, heart rate, and physical activity. RI, resistive index; CKD, chronic kidney disease; CKD, chronic kidney disease; BMI, body mass index; PWV, pulse wave velocity; KES, knee extensor muscle strength; GS, gait speed; 30s-CST, 30 s chair stand test. F statistics value was evaluated by two-way analysis of covariance. * *p* < 0.05 vs. CKD group with high lower extremity muscle strength and function; ^†^ *p* < 0.05 vs. non-CKD group with low lower extremity muscle strength and function; ^§^ *p* < 0.05 vs. non-CKD group with high lower extremity muscle strength and function.

A two-way ANCOVA was used to compare renal RI according to CKD status and lower extremity muscle strength status, with adjustment for the same covariates as in the multiple linear regression models (Figure 3). The two-way ANCOVA showed an interaction effect for KES score and CKD status (Figure 3A), but not for GS and 30s-CST scores (Figure 3B,C). However, some results showed significant main effects for CKD status and/or each lower extremity muscle strength measure. Specifically, individuals with CKD who had a lower KES score had a higher renal RI than those with CKD who had a higher KES score and those without CKD who had a lower KES score. Individuals with CKD who had a lower GS score had a higher renal RI than those without CKD who had a lower GS score. Moreover, individuals with CKD who had a higher GS score had a higher renal RI than those without CKD who had a higher GS score. Individuals with CKD who had a lower 30s-CST score had a higher renal RI than those with CKD who had a higher 30s-CST score and those without CKD who had a lower 30s-CST score.

## 4. Discussion

This cross-sectional study examined the association of lower extremity muscle strength and function with renal flow pulsatility in individuals with and without CKD. GS and 30s-CST scores showed independent negative correlations with renal RI. Individuals with CKD who had lower KES, GS, and 30s-CST scores had the highest renal RI, compared with other participants. These findings suggest that lower extremity muscle strength and function may be potential determinants of renal flow pulsatility in individuals with and without CKD.

It has been well-established that age and central hemodynamics are closely related to renal flow pulsatility [13,14]. Age, aortic pulse pressure, and carotid-femoral PWV were positively correlated with renal RI in the present study (Table 3). Therefore, we performed multiple linear regression analyses to adjust for potential covariates of renal flow pulsatility, including age and central hemodynamic parameters (Table 4, Table 5 and Table 6). The results showed that the KES score was not significantly associated with renal RI. In contrast, GS and 30s-CST scores were significantly associated with renal RI after adjusting for covariates in all participants, those without CKD, and those with CKD. Among the central hemodynamic indices, aortic pulse pressure was shown to be independently associated with renal RI. In a previous study, among the central hemodynamic indices, aortic pulse pressure was shown to be strongly related to renal RI [14]. Therefore, in the multiple regression analysis of this study, we considered that renal RI was significantly associated with aortic pulse pressure rather than hypertension or carotid-femoral PWV. These results suggest mechanistic links between renal flow pulsatility, age, aortic pulse pressure, and lower extremity muscle strength and function.

The increase in renal flow pulsatility with aging and arteriosclerosis confirmed before renal function decline is caused by decreased eGFR. Therefore, individuals with CKD have a higher renal RI than those without CKD [36]. In addition, inspired by our previous study showing that participants with low handgrip strength have high renal RI [15], we hypothesized that individuals with CKD who have decreased lower extremity muscular strength and function would have a higher renal flow pulsatility than those without CKD. Therefore, a two-way ANCOVA was performed to examine the effects of CKD status, lower extremity muscle strength level, and their interaction on renal flow pulsatility. Although a statistically significant interaction was observed only for KES, individuals with CKD who had reduced lower extremity muscle strength and function consistently had high renal RI. Renal RI was comparable between the decreased and increased lower extremity muscle strength groups in individuals without CKD; however, among individuals with CKD, those who had low KES or 30s-CST scores had a higher renal RI than did those who had high KES or 30s-CST scores. These results indicate that renal flow pulsatility may be more strongly affected by lower extremity muscle weakness in individuals with CKD than in those without.

Sarcopenia, defined as loss of muscle mass and decreased muscle strength, is widely known to be associated with vascular damage. This association involves multifactorial mechanisms such as chronic inflammation, oxidative stress, nutrition, and insulin regulation [37,38,39,40]. Furthermore, it has been shown that myokines secreted by muscle may be associated with the inhibition of vascular aging (e.g., arterial stiffness) [41,42]. Hanatani et al. reported that skeletal muscle growth in skeletal muscle-specific Akt1 transgenic mice suppressed renal injury, and these changes were accompanied by increased endothelial nitric oxide synthase (eNOS) phosphorylation in the kidneys [43]. Therefore, muscle mass loss is thought to contribute to increased renal flow pulsatility by decreasing eNOS signaling in the kidneys. Since the lower extremity muscles comprise the majority of the entire body’s muscles (approximately 60–70%), the mechanism reported above may be partially involved in the association of lower extremity muscle strength and function with renal flow pulsatility. However, it should be noted that the KES score was not significantly associated with renal RI in the multiple regression analyses. The inconsistent results may depend on residual confounding such as nutrition and inflammatory status, non-linear relationship of renal RI with KES, and limited sample size. Future studies should confirm the negative association between KES and renal RI while overcoming these methodological weaknesses.

Our previous study showed that participants with low handgrip strength have high renal RI [15]. This study suggests that reduced lower extremity muscle strength and function determines high renal flow pulsatility. Based on the results of this study, it is difficult to conclude which muscle strength is more important in renal RI. However, a previous study of participants with stage 2–4 CKD reported that measures of lower extremity performance were more than 30% lower than predicted, but handgrip strength was relatively preserved [44]. In addition, among the physical functions, impaired lower extremity performance is common in patients with CKD and has been reported to be strongly associated with all-cause mortality [44]. Therefore, we consider lower extremity muscle strength and function to be more related to renal flow pulsatility compared with upper extremity muscle strength.

Lower extremity muscle strength and function are essential in maintaining activities of daily living (ADL) and quality of life (QOL), and these declines are among the symptoms of sarcopenia [21]. Considering the results of this study, strategies to maintain lower extremity muscle strength may be effective in preserving not only ADL and QOL, but also low renal flow pulsatility. The effectiveness of resistance training in maintaining lower extremity muscle strength has been reported in several studies of individuals with and without CKD [45,46]. In a 12-week resistance training intervention study, the control group reported decreased renal function, whereas the resistance training group reported slight improvements in renal function [47]. Future studies are needed to determine whether an intervention to maintain lower extremity muscle strength (e.g., resistance training) is effective in preventing increased renal flow pulsatility.

This study has several limitations. First, we could not identify causal links between renal flow pulsatility and lower extremity muscle strength and function because of the cross-sectional study design. Second, the sample size was relatively small, and there was a difference in the number of participants between individuals with and without CKD. Third, measurement errors may have occurred because the trunk, lower back, and other factors were not adequately fixed in the measurement of KES. However, handheld dynamometers are widely used as a relatively simple and easy way to quantitatively measure lower extremity muscle strength [48]. Fourth, this study did not perform the skeletal muscle mass assessments (e.g., the dual-energy X-ray absorptiometry method). It should be noted that the confirmed negative associations of renal RI with GS and 30s-CST were not adjusted for skeletal muscle mass. Fifth, physical activity was evaluated using a questionnaire rather than an accelerometer, which can be evaluated objectively. Finally, we cannot deny the presence of residual confounding such as nutrition and inflammatory status [37,38,39,40]. Future longitudinal or interventional studies are needed to increase the number of participants and further examine the association of lower extremity muscle strength and function with renal flow pulsatility.

## 5. Conclusions

The present study showed that GS and 30s-CST scores were significantly associated with renal RI in individuals with and without CKD. Furthermore, renal RI was particularly elevated in individuals with CKD who had reduced lower extremity muscle strength and function. The findings of this study have raised the possibility that maintenance of lower extremity muscle strength and function may be important in preventing elevated renal flow pulsatility, particularly in individuals with CKD.

## Figures and Tables

**Table 1:** Characteristics of the participants.

Variable	All (n = 277)	Non-CKD (n = 176)	CKD (n = 101)	*p*
Age, years	64 ± 9	63 ± 9	66 ± 8	0.005
Women, n (%)	184 (66)	132 (75)	52 (51)	<0.001
Height, cm	159.2 ± 8.4	158.7 ± 7.9	160.0 ± 9.2	0.255
Body mass, kg	57.1 ± 10.9	55.8 ± 10.2	59.3 ± 11.9	0.014
BMI, kg/m^2^	22.4 ± 3.3	22.1 ± 3.0	23.1 ± 3.8	0.052
eGFR, mL/min/1.73 m^2^	82 [64–95]	90 [81–97]	57 [45–74]	<0.001
Urinary ACR, mg/g	14 [7–38]	9 [6–14]	76 [32–446]	<0.001
Heart rate, bpm	60 [54–65]	59 [54–63]	60 [54–65]	0.002
Brachial systolic blood pressure, mmHg	122 ± 15	119 ± 14	128 ± 15	<0.001
Brachial diastolic blood pressure, mmHg	73 ± 10	71 ± 10	77 ± 10	<0.001
Mean arterial pressure, mmHg	90 ± 11	87 ± 11	94 ± 11	<0.001
Aortic pulse pressure, mmHg	38 ± 8	37 ± 8	40 ± 8	0.001
Carotid-femoral PWV, cm/s	900 [786–1029]	851 [759–956]	1007 [904–1161]	<0.001
Renal RI	0.63 ± 0.06	0.61 ± 0.05	0.65 ± 0.06	<0.001
Renal RI ≥ 0.70, n (%)	21 (8)	5 (3)	16 (16)	<0.001
Knee extensor muscle strength, kgf/kg	0.60 ± 0.17	0.59 ± 0.16	0.60 ± 0.19	0.662
Gait speed, m/s	2.35 ± 0.45	2.42 ± 0.45	2.23 ± 0.44	<0.001
30 s chair stand test, repetitions	20 ± 5 *	21 ± 5 †	18 ± 5	<0.001
Physical activity, METs- hour/week	1386 [594–2772]	1386 [588–2772]	1386 [594–2772]	0.971
GFR stages				<0.001
eGFR stage 1 (eGFR: ≥90 mL/min/1.73 m^2^), n (%)	101 (37)	90 (51)	11 (11)	
eGFR stage 2 (eGFR: 60–89 mL/min/1.73 m^2^), n (%)	117 (42)	86 (49)	31 (31)	
eGFR stage 3 (eGFR: 30–59 mL/min/1.73 m^2^), n (%)	50 (18)	0 (0)	50 (49)	
eGFR stage 4 (eGFR: 15–29 mL/min/1.73 m^2^), n (%)	9 (3)	0 (0)	9 (9)	
Albuminuria stages				<0.001
Normoalbuminuria (urinary ACR: <30 mg/g), n (%)	194 (70)	176 (0)	18 (18)	
Microalbuminuria (urinary ACR: 30–299 mg/g), n (%)	51 (18)	0 (0)	51 (50)	
Macroalbuminuria (urinary ACR: ≥300 mg/g), n (%)	32 (12)	0 (0)	32 (32)	
Antihypertensive medicine, n (%)	89 (32)	25 (14)	64 (63)	<0.001
Hypoglycemic medicine, n (%)	19 (7)	4 (2)	15 (15)	<0.001
Antidyslipidemic medicine, n (%)	70 (25)	25 (14)	45 (45)	<0.001
Hypertension, n (%)	108 (39)	36 (20)	72 (71)	<0.001
Diabetes, n (%)	63 (23)	27 (15)	36 (36)	<0.001
Dyslipidemia, n (%)	150 (54)	89 (51)	61 (60)	0.114

Data are shown as the mean ± standard deviation, median [interquartile range], or number (%). * n = 276. ^†^ n = 175. CKD, chronic kidney disease; BMI, body mass index; eGFR, estimated glomerular filtration rate; ACR, albumin creatinine ratio; PWV, pulse wave velocity; RI, resistive index.

**Table 2:** Renal RI in individuals with and without CKD by GFR stage.

	Non-CKD	CKD	*p*
eGFR stage 1 (Non-CKD, n = 90; CKD, n = 11)			
Renal RI	0.60 ± 0.04	0.64 ± 0.05	0.032
eGFR stage 2 (Non-CKD, n = 86; CKD, n = 31)			
Renal RI	0.63 ± 0.05	0.64 ± 0.05	0.161

**Table 3:** Simple correlation matrix for renal RI.

	All		Non-CKD		CKD	
	*r*	*p*	*r*	*p*	*r*	*p*
Age	0.484	<0.001	0.562	<0.001	0.325	<0.001
BMI	0.182	0.002	0.060	0.426	0.234	0.019
eGFR	−0.422	<0.001	−0.278	<0.001	−0.272	0.006
Urinary ACR	0.308	<0.001	0.286	<0.001	0.245	0.014
Heart rate	−0.007	0.911	−0.066	0.384	−0.101	0.313
Brachial SBP	0.298	<0.001	0.248	<0.001	0.166	0.097
Brachial DBP	−0.052	0.392	−0.085	0.261	−0.288	0.004
Mean arterial pressure	0.104	0.085	0.060	0.430	−0.100	0.321
Aortic pulse pressure	0.566	<0.001	0.527	<0.001	0.561	<0.001
Carotid-femoral PWV	0.457	<0.001	0.327	<0.001	0.424	<0.001
KES	−0.132	0.028	−0.090	0.236	−0.221	0.027
GS	−0.329	<0.001	−0.335	<0.001	−0.208	0.037
30s-CST * ^†^	−0.307	<0.001	−0.206	0.006	−0.327	<0.001
Physical activity	0.023	0.702	−0.044	0.564	0.157	0.116

Univariate analyses were performed using Pearson’s correlation coefficients. RI, resistive index; CKD, chronic kidney disease; BMI, body mass index; eGFR, estimated glomerular filtration rate; ACR, albumin creatinine ratio; SBP, systolic blood pressure; DBP, diastolic blood pressure; PWV, pulse wave velocity; KES, knee extensor muscle strength; GS, gait speed; 30s-CST, 30 s chair stand test. * All participants (n = 276). ^†^ Non-CKD adults (n = 175).

**Table 4:** Multivariable-adjusted linear regression models for renal RI in all participants.

	Model 1		Model 2		Model 3 *	
	(R^2^ = 0.558, *p* < 0.001)		(R^2^ = 0.569, *p* < 0.001)		(R^2^ = 0.578, *p* < 0.001)	
	*β*	*p*	*β*	*p*	*β*	*p*
KES, kgf/kg	−0.003	0.952	−	−	−	−
GS, m/s	−	−	−0.115	0.012	−	−
30s-CST, repetitions	−	−	−	−	−0.153	<0.001
Age, years	0.281	<0.001	0.248	<0.001	0.273	<0.001
Sex (1: women)	0.009	0.863	−0.024	0.640	0.005	0.913
BMI, kg/m^2^	0.044	0.353	0.031	0.505	0.021	0.652
eGFR, mL/min/1.73 m^2^	−0.227	<0.001	−0.215	<0.001	−0.191	0.001
Urinary ACR, mg/g	0.096	0.067	0.086	0.101	0.108	0.037
Hypertension (1: yes)	−0.073	0.162	−0.068	0.188	−0.063	0.218
Diabetes (1: yes)	0.215	<0.001	0.216	<0.001	0.224	<0.001
Dyslipidemia (1: yes)	−0.007	0.877	−0.004	0.926	−0.015	0.738
Physical activity, METs-hour/week	−0.058	0.172	−0.055	0.192	−0.045	0.285
Heart rate, bpm	−0.021	0.665	−0.028	0.566	−0.043	0.369
Aortic pulse pressure, mmHg	0.383	<0.001	0.368	<0.001	0.360	<0.001
Carotid-femoral PWV, cm/s	0.028	0.633	0.030	0.597	0.029	0.608

*β*: standardized regression coefficients. RI, resistive index; KES, knee extensor muscle strength; GS, gait speed; 30s-CST, 30 s chair stand test; BMI, body mass index; eGFR, estimated glomerular filtration rate; ACR, albumin creatinine ratio; PWV, pulse wave velocity. * n = 276.

**Table 5:** Multivariable-adjusted linear regression models for renal RI in individuals without CKD.

	Model 1		Model 2		Model 3 ^†^	
	(R^2^ = 0.485, *p* < 0.001)		(R^2^ = 0.491, *p* < 0.001)		(R^2^ = 0.491, *p* < 0.001)	
	*β*	*p*	*β*	*p*	*β*	*p*
KES, kgf/kg	0.097	0.129	−	−	−	−
GS, m/s	−	−	−0.133	0.042	−	−
30s-CST, repetitions	−	−	−	−	−0.124	0.046
Age, years	0.412	<0.001	0.363	<0.001	0.391	<0.001
Sex (1: women)	−0.011	0.876	−0.059	0.420	−0.035	0.623
BMI, kg/m^2^	0.092	0.178	0.038	0.566	0.027	0.687
eGFR, mL/min/1.73 m^2^	−0.058	0.416	−0.092	0.191	−0.074	0.291
Urinary ACR, mg/g	0.064	0.351	0.034	0.612	0.038	0.575
Hypertension (1: yes)	−0.103	0.126	−0.077	0.255	−0.080	0.236
Diabetes (1: yes)	0.074	0.223	0.072	0.234	0.077	0.204
Dyslipidemia (1: yes)	0.045	0.461	0.039	0.527	0.033	0.592
Physical activity, METs- hour/week	−0.110	0.067	−0.098	0.097	−0.091	0.124
Heart rate, bpm	−0.047	0.481	−0.042	0.534	−0.071	0.301
Aortic pulse pressure, mmHg	0.371	<0.001	0.342	<0.001	0.347	<0.001
Carotid-femoral PWV, cm/s	−0.001	0.988	−0.016	0.835	0.001	0.992

*β*: standardized regression coefficients. RI, resistive index; CKD, chronic kidney disease; KES, knee extensor muscle strength; GS, gait speed; 30s-CST, 30 s chair stand test; BMI, body mass index; eGFR, estimated glomerular filtration rate; ACR, albumin creatinine ratio; PWV, pulse wave velocity. ^†^ n = 175.

**Table 6:** Multivariable-adjusted linear regression models for renal RI in individuals with CKD.

	Model 1		Model 2		Model 3	
	(R^2^ = 0.596, *p* < 0.001)		(R^2^ = 0.605, *p* < 0.001)		(R^2^ = 0.630, *p* < 0.001)	
	*β*	*p*	*β*	*p*	*β*	*p*
KES, kgf/kg	−0.093	0.235	−	−	−	−
GS, m/s	−	−	−0.149	0.069	−	−
30s-CST, repetitions	−	−	−	−	−0.222	0.003
Age, years	0.228	0.005	0.186	0.028	0.202	0.010
Sex (1: women)	−0.033	0.715	−0.049	0.579	0.024	0.767
BMI, kg/m^2^	0.019	0.816	0.005	0.949	0.026	0.741
eGFR, mL/min/1.73 m^2^	−0.230	0.019	−0.198	0.047	−0.164	0.088
Urinary ACR, mg/g	0.062	0.479	0.066	0.442	0.118	0.168
Hypertension (1: yes)	0.002	0.985	0.019	0.811	0.017	0.823
Diabetes (1: yes)	0.349	<0.001	0.377	<0.001	0.370	<0.001
Dyslipidemia (1: yes)	−0.046	0.569	−0.037	0.643	−0.078	0.306
Physical activity, METs- hour/week	−0.009	0.909	−0.021	0.780	0.003	0.962
Heart rate, bpm	−0.008	0.923	−0.028	0.738	−0.017	0.833
Aortic pulse pressure, mmHg	0.439	<0.001	0.429	<0.001	0.400	<0.001
Carotid-femoral PWV, cm/s	0.043	0.641	0.069	0.460	0.052	0.557

*β*: standardized regression coefficients. RI, resistive index; CKD, chronic kidney disease; KES, knee extensor muscle strength; GS, gait speed; 30s-CST, 30 s chair stand test; BMI, body mass index; eGFR, estimated glomerular filtration rate; ACR, albumin creatinine ratio; PWV, pulse wave velocity.

## Data Availability

The data presented in this study are available upon request from the corresponding author. The data were not publicly available due to privacy concerns.
